# A biomechanical cause of low power production during FES cycling of subjects with SCI

**DOI:** 10.1186/1743-0003-11-123

**Published:** 2014-08-16

**Authors:** Johann Szecsi, Andreas Straube, Che Fornusek

**Affiliations:** Department of Neurology, Center for Sensorimotor Research, Ludwig-Maximilians University, Marchioninistrasse 23, Munich, 81377 Germany; Exercise, Health and Performance Faculty Research Group, Faculty of Health Sciences, University of Sydney, Marchioninistrasse 23, Munich, Sydney, Australia

**Keywords:** Generalised muscle moment, Joint power, Electrical stimulation, Cycling, Spinal cord injury, Rehabilitation

## Abstract

**Background:**

The goal of Functional Electrical Stimulation (FES) cycling is to provide the health benefits of exercise to persons with paralysis. To achieve the greatest health advantages, patients should produce the highest possible mechanical power. However, the mechanical power output (PO) produced during FES cycling is very low. Unfavorable biomechanics is one of the important factors reducing PO. The purpose of this study was to investigate the primary joints and muscles responsible for power generation and the role of antagonistic co-contraction in FES cycling.

**Methods:**

Sixteen subjects with complete spinal cord injury (SCI) pedaled a stationary recumbent FES tricycle at 60 rpm and a workload of 15 W per leg, while pedal forces and crank angle were recorded. The joint muscle moments, power and work were calculated using inverse dynamics equations.

**Results:**

Two characteristic patterns were found; in 12 subjects most work was generated by the knee extensors in the propulsion phase (83% of total work), while in 4 subjects most work was shared between by the knee extensors (42%) and flexors (44%), respectively during propulsive and recovery phases. Hip extensors produced only low net work (12 & 7%). For both patterns, extra concentric work was necessary to overcome considerable eccentric work (-82 & -96%).

**Conclusions:**

The primary power sources were the knee extensors of the quadriceps and the knee flexors of the hamstrings. The antagonistic activity was generally low in subjects with SCI because of the weakness of the hamstrings (compared to quadriceps) and the superficial and insufficient hamstring mass activation with FES.

**Electronic supplementary material:**

The online version of this article (doi:10.1186/1743-0003-11-123) contains supplementary material, which is available to authorized users.

## Background

Functional electrical stimulation (FES) propelled leg cycling is an established method of exercising the legs of persons with SCI to bestow both central and peripheral health benefits. Previous research has demonstrated that in SCI individuals FES cycling can improve cardiovascular and respiratory function [1–4], body composition [5], muscle mass [6], bone mass [7], and quality of life [8]. FES cycling can also be used for outdoor cycling recreation and mobility [9, 10].

The mechanical power output (PO) produced during FES cycling is very low (i.e. 8–35 W) [11] which is an order of magnitude lower than power obtained in volitional cycling of able-bodied (AB) persons. However, the health benefits bestowed by FES cycling are strongly related to the PO that can be generated [1, 11–14]. Likewise, the PO is too low for outdoor cycling except for on short horizontal tracks [9, 11, 13, 15], thus limiting motivation and enjoyment. Therefore, the goal is to understand the reason for the difference between volitional AB cycling and FES cycling in persons with SCI. Understanding may allow us to modify FES cycling to improve the PO produced.

Three factors are thought responsible for the lower power outputs achieved with FES cycling in persons with SCI: 1) the inefficiency of artificial muscle activation, 2) the crude control of muscle groups accomplished by stimulation, and 3) muscle atrophy and transformation due to chronic paralysis and disuse. All these causes also lead to an increased fatigue rate, further limiting the health benefits [5] of the workout [16, 17].

The surface electrical stimulation allows only crude control over which muscle groups contract. This could lead to imprecise flexor and extensor coordination and result in less efficient cycling biomechanics. This biomechanical inefficiency has previously been suggested to be the most important factor contributing to low cycling power production [13, 18]. Compared to normal voluntary motor control activation, electrical stimulation control is much cruder and coarsely recruits groups of mono- and bi-articular muscles (e.g. quadriceps, hamstrings, or gluteal muscle groups). Thus joint moment and power distribution with FES are likely different to normal activation, reducing the efficiency of movement. For example, the crude activation strategy of muscle groups with FES-cycling may result in more antagonistic flexor/extensor co-contractions producing more concentric (positive) and eccentric (negative) power across the joints [15] than with volitional cycling. Because only the net work difference contributes to the external work output produced at the pedal crank over a revolution, it is assumed that more metabolic energy would be consumed with FES cycling [13] for a given mean PO (Additional file 1: Appendix 1b-c). Thus to understand the low power production in individuals with SCI performing FES-cycling, the flexor/extensor moment patterns (including co-contractions) and the concentric/eccentric power patterns of the muscles across the joints must be known [19].

Few published studies have described joint power generation during volitional recumbent cycling of AB subjects. An early study [20] conducted at high workload (250 W) demonstrated, that similar to upright cycling, power was produced during recumbent cycling by concentric muscle work mainly by the knee (55%) and hip extensors and flexors (25%), in a fairly balanced manner. A recent study [21] that investigated AB subjects performing volitional cycling at low workload (30 W), supported these findings by showing that power was mainly concentric and approximately similarly distributed between the knee (57%) and the hip (43%) extensors and flexors.

However, contrary to the data from AB subjects cycling, the study on FES- supported outdoor cycling of subjects with SCI [15] indicated that the preponderant part of the total work was generated by concentrically activated knee extensors alone, with a considerable excess being absorbed by the eccentrically activated hip flexors. In contrast, two other studies on SCI FES cycling suggested, that hip joint extensors may provide the majority of work to the crank [19, 22]. In conclusion muscle and joint power contribution during FES cycling are hitherto poorly understood.

The purpose of this study was to investigate the primary joints and muscles responsible for power generation by measuring the joint muscle moment and power patterns during SCI FES propelled cycling. A second purpose of this study was to investigate the degree and role of antagonist co-contractions induced by the muscle stimulation. Such information will be useful for better understanding and future optimization of the biomechanical efficiency of FES cycling in subjects with SCI.

## Methods

### Subjects

Sixteen healthy subjects (5 women, 11 men; 42.3 [9.4] y old; mean[SD]) with chronic (10.8 [6.0] y since injury) and motor complete spastic SCI (ASIA-A) at the level between the C5 and T12 vertebra participated in the study. The height and body-mass of the subjects was 1.79 (0.10) m and 77.0 (11.9) kg, respectively. Fourteen subjects showed no or low levels of muscle spasm (Modified Ashworth Scale [MAS] 0–1), one subject showed moderate extension spasm (MAS 2) and one subject showed high flexion/extension spasm (MAS 3–4) at the beginning of the measurements. The subjects were able to comprehend commands and had experience with FES cycling. Each subject had performed home training with his/her ergometer 1–3 times per week for between 0.5-4 years. To be included participants had to be able to cycle at a workload of 30 W (both legs) for at least 1 minute. The University of Munich ethics committee approved the study. All subjects gave their informed consent before participation.

### Equipment

A stationary tricycle with its front wheel replaced by a servomotor axle (AC-servo MR 7434, ESR Pollmeier Ltd, Ober-Ramstadt, Germany) with cadence and resistance moment control served as a test-bed for the trials [21]. Each lower leg was inserted into a pedal boot that was fixed to the pedal. The pedal boot orthosis held the ankle joint at 90° and restricted leg movement to the sagittal plane. An 11-bit incremental encoder determined the crank position. The tangential and radial forces applied to the both cranks were collected simultaneously by instrumented crank arms (length 0.15 m) that acquired the tangential and radial force components via two Hall sensors (o-tec Ltd., Bensheim, Germany). The Hall sensors were calibrated with 5 different weights (20.6-, 31.7-, 63.4-, 126.8- and 253.6-N) achieving a linearity corresponding to R^2^ = 0.98. Pedal forces could be measured in both directions to an accuracy of ±1.8 N. Custom written software on a PC collected pedal force and crank angle data at 1000 Hz via a 32-channel 12-bit resolution analog data acquisition card. The PC controlled the muscle stimulator via a USB interface. Pedaling cadence was maintained between 57–63 rpm by modulation of the stimulation intensity delivered to the muscles, using an incremental controller with 2 mA steps [23].

### Stimulation

The quadriceps and hamstrings muscle groups were stimulated [24, 25] for cycling. The gluteal muscles were not stimulated because they produced no measurable crank torques in most subjects. A constant-current stimulator (Hasomed GmbH, D-39114 Magdeburg, Germany) provided the stimulation current (rectangular, biphasic, pulse width 500 μs, maximal pulse amplitude 127 mA, and 30 Hz frequency) [26, 27]. Pairs of self-adhesive gel electrodes (4.5 cm × 9.5 cm) were used. For the quadriceps, the proximal electrode centre was placed on the skin over the motor point at approximately 1/3 of the distance from the inguinal line to the superior patellar border and the distal electrode centre was placed 6–8 cm proximally to the patellar border. For the hamstrings the proximal electrode centre was placed 2–4 cm below the gluteal crease and the distal electrode centre was placed above 4–5 cm above the popliteal space [28].

Muscle stimulation crank angle (firing) ranges [29] were individually determined by preliminary isometric crank torque measurements (Additional file 1: Appendix 2). Each muscle was stimulated 50° earlier in the crank revolution to compensate for a muscle force rise time of ~140 ms at a cadence of 60 rpm.

### Measurement protocol

Each participant’s anthropometric data (height, weight) and their tricycle seat position (hip joint to crank axle distance and inclination) were recorded. The seat position was adjusted so that the knee extension did not exceed 150-160° at the bottom dead centre. Stimulated cycling was performed for one minute at 60 rpm cadence, to achieve maximum PO [30]. Thus each subject performed a trial of 90 s consisting of 15 s of passive cycling (machine driven leg turning), 60 s of FES-driven active pedaling, and a second 15 s passive cycling period. The passive cycling was performed at a cadence of 60 rpm set by the servomotor (isokinetic cycling). The crank moments (calculated as sum of tangential pedal forces times crank arm) were recorded during the first passive cycling period and used to calculate the mean passive moment over one crank turn cycle.

During the active period the subjects pedaled with FES against the machine controlled resistance (isotonic cycling). To set the target workload to 30 W (15 W per leg), which was established in preliminary measurements such that it was maintainable for at least 1 min for all selected subjects, the resistance was set to 4.9 Nm + the mean passive moment from the preceding passive period (1rev/s × 2π × 4.9 Nm ≈ 30 W). During active cycling the cadence of 60 rpm was maintained by automatic modulation of the stimulation pulse amplitude. The last 15 s of the active stimulated cycling period were recorded for data analysis (active pedal forces and kinematics). Data from the following 15 s of passive cycling were also recorded (passive pedal forces and kinematics).

### Offline data processing

The crank angle data were low-pass zero-lag filtered with 5 Hz cut-off capturing 97-93% of the signal power [31] before obtaining cadence and acceleration. Software was used to separate each trial into individual revolutions. Fifteen revolutions (15 s of data) were then ensemble-averaged together to obtain a representative revolution for each trial.

Knee- and hip-joint moments of the right leg were obtained by inverse dynamic analysis [32, 33] whereas the right lower limbs were modeled as planar, two-segment, rigid body systems with external reaction forces at the pedal spindles (Additional file 1: Appendix 1a). Leg segment kinematics, tangential and radial pedal forces, body segment parameters [34], and seat position served as inputs to the inverse dynamic analysis [15] using the SimMechanics Toolbox (MATLAB 7.12, MathWorks, Natick, MA, USA).

The inverse dynamics approach calculates joint moments assuming ideal frictionless solid bodies with the hip joint rotation axes fixed in space. The joint moments calculated during active cycling are the FES evoked muscle moments that drive both the legs and the cycle test-bed system. During passive cycling, zero driving moment should occur on the joints from the muscles. However, during passive cycling the motor drives the legs against the small but nonzero joint moments (passive joint moments) caused by muscle spasticity, ligament or capsule elastic [35] or viscous [36] joint forces. Passive joint moments could also include error contributions caused by assumptions concerning the rigidity of links and the fixed position of the hip joint axes [37]. In the current study which uses low workloads, the passive joint moment is not negligible compared to the active joint moment [21]. During active FES cycling the muscles drive the legs producing an active joint moment, which also overcomes the passive joint moment. Thus additional to the passive joint moment, the active joint moment also contains the muscle contractile component and a tension dependent muscle elastic component.

**Figure 1:**
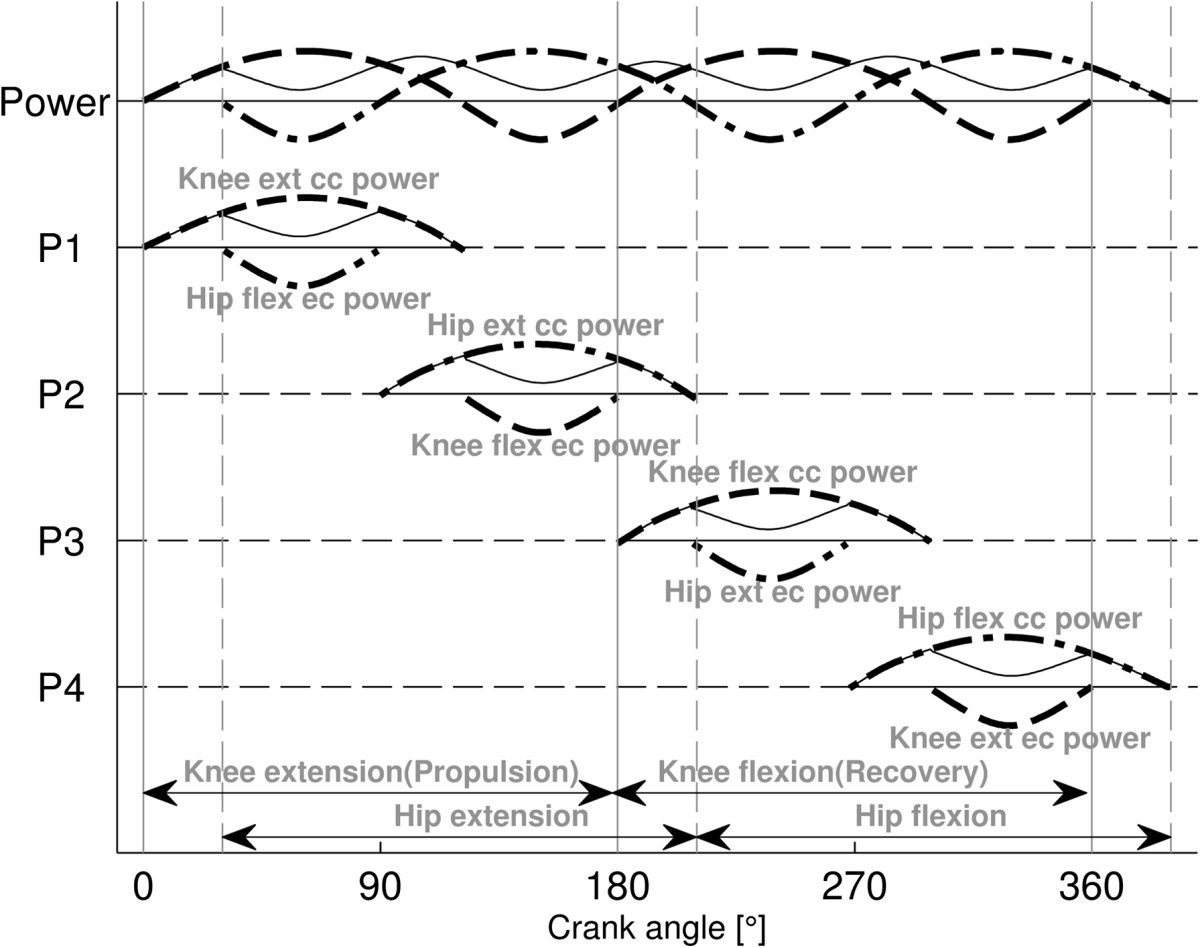
**Schematic graph for definition of the power components P1-P4 contributing toward total power (Power) during a crank revolution.** The first power component (P1) represents concentric knee extensor and eccentric hip flexor power; the second component (P2) represents concentric hip extensor and eccentric knee flexor power; the third component (P3) represents concentric knee flexor and eccentric hip extensor power, and the fourth component (P4) represents concentric hip flexor and eccentric knee extensor power. The knee (dashed), the hip (dashed-dotted) and the net power (continuous thin) are shown. Concentric and eccentric powers are positive and negative, respectively.

Therefore inverse dynamic calculations were performed twice for each subject by the same equations and parameters, using active and passive kinematics and pedal force data, and obtaining active and passive joint moments, power and work, respectively. Subsequently, to accurately calculate the net active joint moment (joint moment), we subtracted the passive cycling joint moment from the active joint moment obtained from FES propelled cycling. Subtraction was performed by relating the moments to the crank revolution cycle. Joint power was calculated by multiplying the joint moment by the cadence (corresponding to active cycling). Mean joint moments were slightly adjusted to achieve 15 W one-sided power, respectively.For each subject the joint moment and power were referred to the revolution cycle of the crank, which was defined as being composed of a knee extension phase followed by a knee flexion phase (Figure 1). Accordingly, propulsion and recovery phases were defined as knee extension and flexion phases, respectively (top dead centre TDC = 0° and bottom dead centre BDC = 180° crank angle).

Former theoretical and experimental work based on inverse dynamic analysis has shown that for both upright [31] and recumbent cycling of AB subjects [21], the joint extensor and flexor moments contribute to total mechanical power throughout the crank cycle sequentially, by generating typically four power components generated by concentric action of the knee extensors, hip extensors, knee flexors and hip flexors, in this order. However because of the action of bi-joint muscle groups, each concentric extensor/flexor action at a joint may be associated with an eccentric flexor/extensor action from the other joint. Thus each power component contains a concentric and an eccentric part, and the net power produced by the component is the difference of the parts (Figure 1).

**Figure 2:**
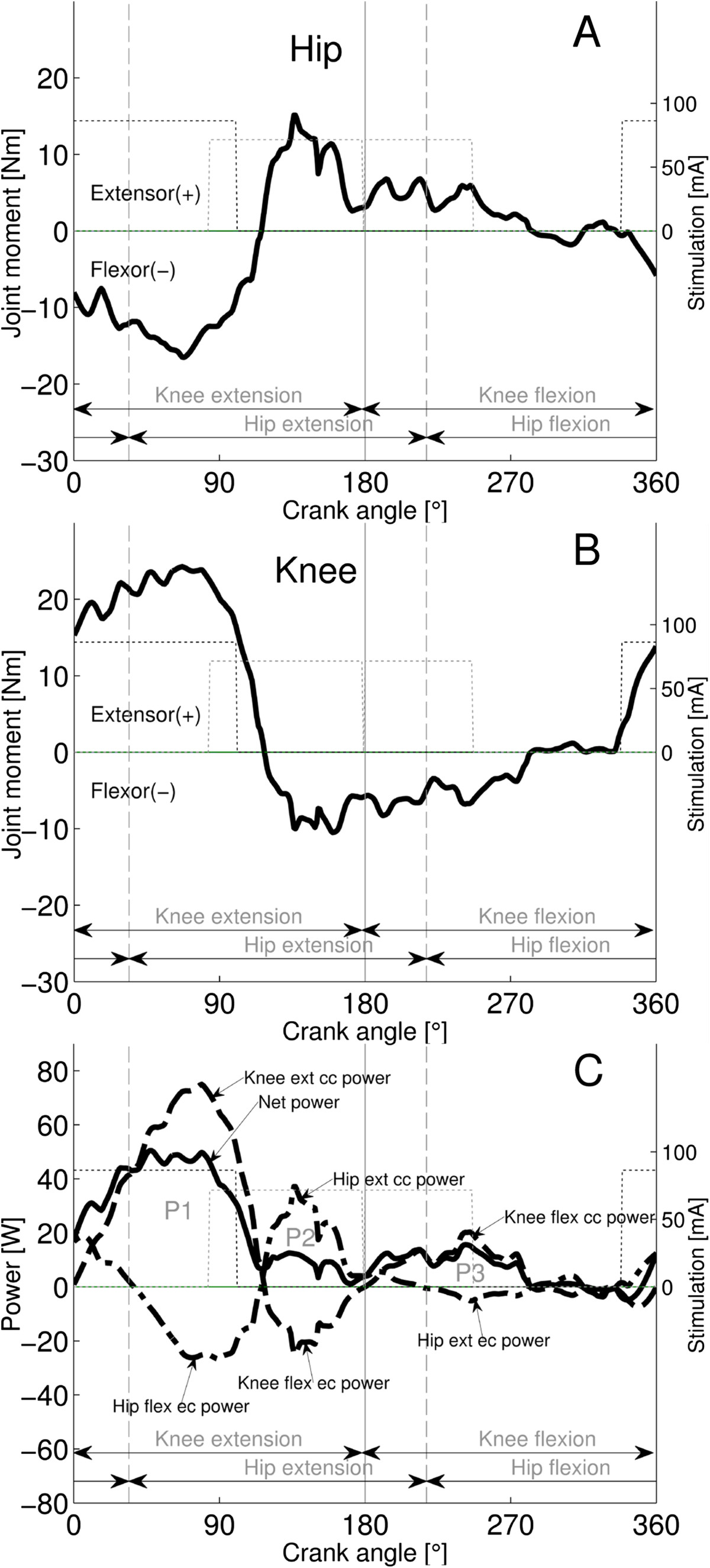
**Joint muscle moments and power produced by the right leg of a representative subject with SCI pedaling at 60 rpm with stimulation of quadriceps and hamstrings to produce 15 W. Panel** **A** and **B**: The hip and knee joint moment patterns. Panel **C**: the knee (dashed), hip (dashed-dotted) and net power (continuous) are shown. The power components P1-P3 are marked in Panel **C** to represent the different contributions to the net power. Phase advance corrected stimulation intensities (on-off step-functions) for quadriceps (thin black dotted) and hamstrings stimulation (thin grey dotted) are shown.

Typically, the first power component (P1) occurs in the early-middle knee-extension phase representing concentric knee extensor and eccentric hip flexor power; the second component (P2) occurs in the middle-late hip-extension phase representing concentric hip extensor and eccentric knee flexor power; the third component (P3) occurs in the early-middle knee-flexor phase representing concentric knee flexor and eccentric hip extensor power. Finally, the fourth component (P4) occurs in the middle-late hip-flexor phase and represents concentric hip flexor and eccentric knee extensor power. While in AB subjects it is problematical to allocate power components to the action of individual muscles or muscle groups, in contrast in subjects with SCI, the power components can be assumed to be produced by specifically stimulated muscle groups (but see Discussion). If there is only a small overlap of the hamstrings and quadriceps stimulation firing ranges (Figure 2), then P1 is mainly produced by the quadriceps and P2 and P3 are mainly produced by the hamstrings contraction (Figure 2C; Additional file 1: Appendix 3). However, if a large overlap exists between the stimulation ranges, then P1 and P2 represent the summed power of the co-contraction of both quadriceps and hamstrings (Additional file 1: Appendix 4).

The concentric (positive), eccentric (negative) and net work were respectively defined as the time integration of concentric and eccentric power, and their algebraic sum. The extension phase work was defined as net P1 work produced during knee extension and net P2 work produced during hip extension phase (=net P1 + net P2). Accordingly, the flexion phase work was the net P3 work produced during knee flexion phase and the net P4 work produced during hip flexion phase (=net P3+ net P4).

### Statistics

Descriptive statistics (mean[SD]) were calculated by averaging the right leg data of the subjects, the cadence, the peak joint moment magnitude and crank angle, the concentric, eccentric, component related, joint related and net work. For graphic representation of the average joint moment and power profiles, mean knee and hip extension/flexion ranges were computed. Extension vs. flexion phase work, concentric vs. net work, and knee vs. total work were compared using paired Wilcoxon tests because normal distribution could not be assumed. The level of statistical significance was p < 0.05.

## Results

### Cadence and power

All 16 subjects successfully completed the measurement procedure. The average workload achieved was 29.9 (1.3) W at cadence 60.3 (2.6) rpm and stimulation intensities 88.4 (28.1) mA and 72.4 (23.6) mA for quadriceps and hamstrings, respectively. Two different patterns of work production were observed; in a majority of subjects the major part of work obtained was extension phase work and only small amounts were flexion phase work, while in the remaining subjects comparable amounts of extension and flexion phase work were generated. To clarify the trends in the data, we separated the patterns by choosing a threshold for the contribution of the flexor phase work. Thus for analysis we separated the subjects into two groups; 'P1P2' group which were those 12 subjects who produced less than 20% of total work as flexion phase work, and 'P1P3' group which consisted of the remaining 4 subjects that produced more than 20% total work as flexion phase work.

### Joint moments

**Figure 3:**
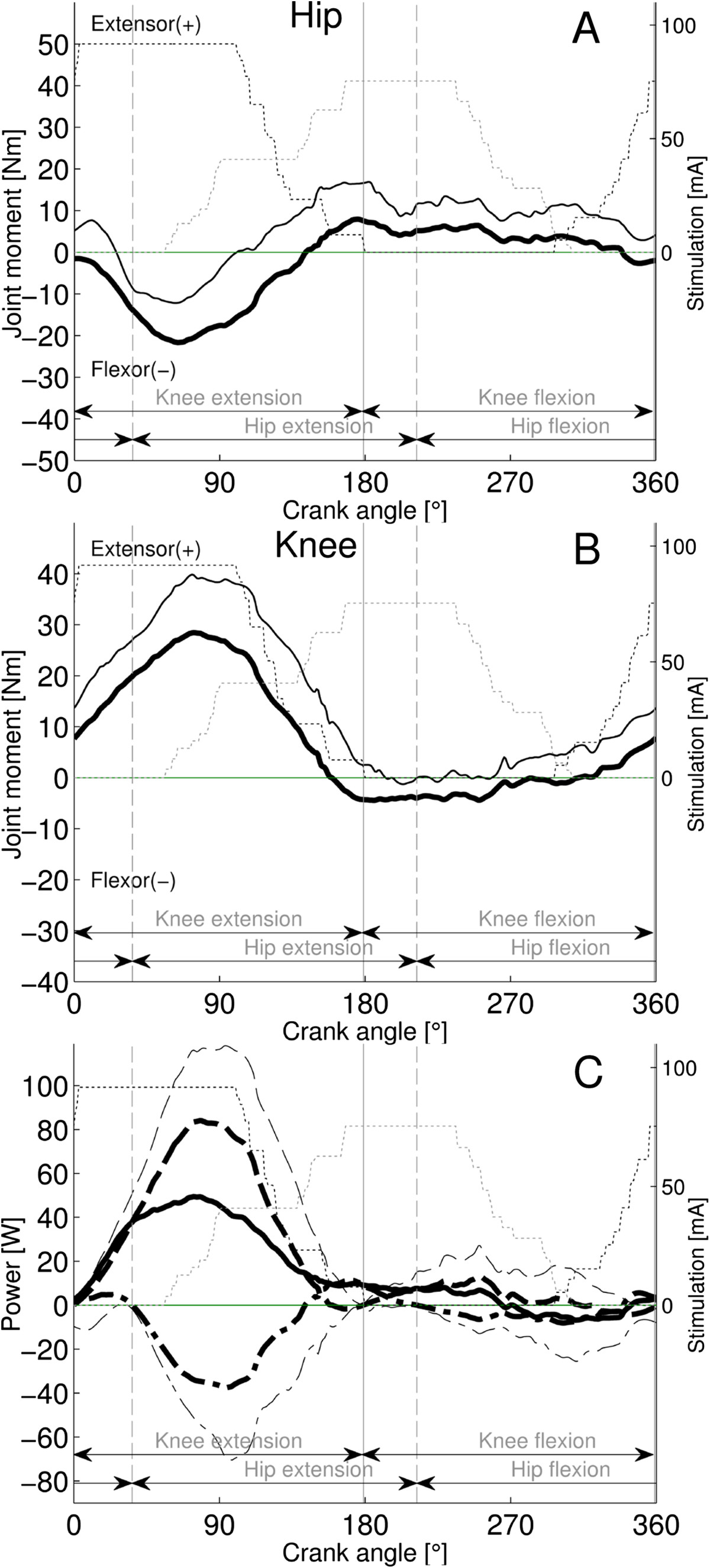
**Joint muscle moments and power for the P1P2 group (n = 12).** The patterns represent means (bold black lines) + SD (thin black lines). Panel **A** and **B**: The hip and knee joint moment patterns. Panel **C**: The knee (mean dashed; SD thin dashed) and hip (mean dashed-dotted; SD thin dashed-dotted) power patterns are displayed. Net power is a continuous black line with no SD displayed. The stepwise patterns displayed for quadriceps (thin black dotted) and hamstrings stimulation (thin grey dotted) intensity resulted from averaging of the on-off step-functions across subjects. The stimulations are phase advance corrected.

**Figure 4:**
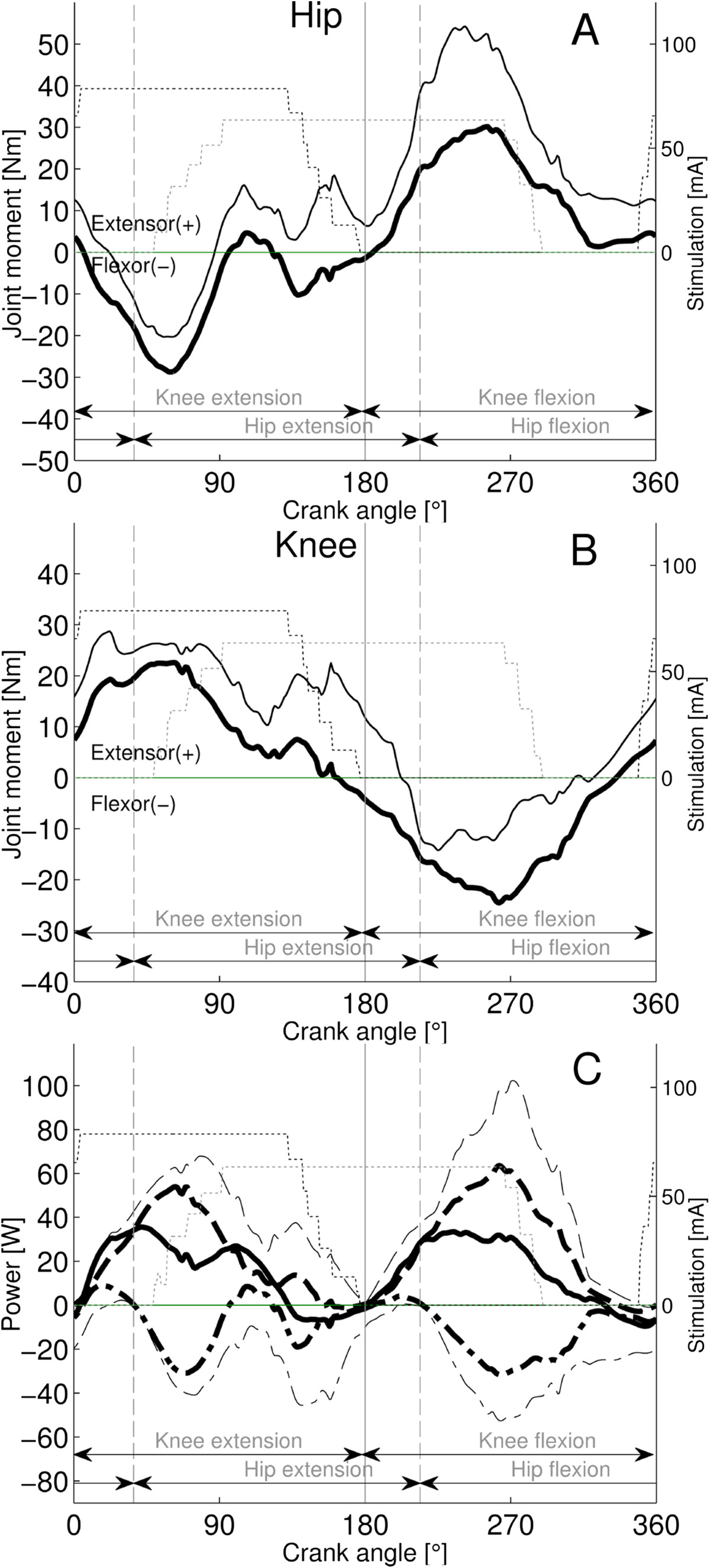
**Joint muscle moments and power for the P1P3 group (n = 4).** The patterns represent means (bold black lines) + SDs (thin black lines). Panel **A** and **B**: The net hip and knee joint moment patterns. Panel **C**: The knee (mean dashed; SD thin dashed) and hip (mean dashed-dotted; SD thin dashed-dotted) power patterns are displayed. Net power is a continuous black line with no SD displayed. The stepwise patterns displayed for quadriceps (thin black dotted) and hamstrings stimulation (thin grey dotted) intensity resulted from averaging of the on-off step-functions across subjects. The stimulations are phase advance corrected.

For both groups P1P2 and P1P3, the knee/hip joint moment patterns showed a biphasic behavior with an extensor/flexor and a flexor/extensor moment activity over most of the propulsion and the recovery phases, respectively (Figure 3A-B, Figure 4A-B). Extensor and flexor moment activity were generally pronounced with prominent peaks (Table 1), however with two exceptions for the P1P2 group; the hip extensor and knee flexor moments were weak over the late propulsion and the recovery phase (Figure 3A-B). Additionally, for the P1P3 group, the hip joint moment oscillated between extensor and flexor during the late propulsion phase.

**Table 1:** **Summary of group averages for knee and hip general muscle moment magnitudes**

		Group	Peak moment [Nm]	Peak moment angle [°]
Knee	Maximum	P1P2	28.5(11.5)	74(55)
		P1P3	22.9(3.9)	58(102)
	Minimum	P1P2	-4.5(6.2)	186(57)
		P1P3	-24.5(12.3)	262(37)
Hip	Maximum	P1P2	7.9(8.5)	176(63)
		P1P3	30.1(21.0)	255(33)
	Minimum	P1P2	-21.7(9.4)	64(60)
		P1P3	-29.3(8.4)	58(44)

Data are presented as average (standard deviation).

### Joint power

For the P1P2 group (Figure 3C) positive net power was generated over the revolution cycle mainly by the power component P1 which resulted from knee extension and hip flexion power. The concentric hip extensor power showed a small peak around BDC, representing a small P2 component. Similarly, small peaks of the concentric knee flexor power at 250° and of the concentric hip flexor power around the TDC relate to small P3 and P4 components. Power components P1, P2, P3 and P4 contributed to net power production with 83%, 12%, 2% and 3%, respectively (Table 2). To produce net positive work (100%), significantly more concentric work (182%) was generated (p < 0.001), while considerable power was absorbed by eccentric contractions. Because of the dominance of the P1 component, the knee joint generated overall positive work, which was significantly more (145%, p <0.01) than the net work obtained (100%), and the hip joint absorbed the excess. As the P1 component was localized in the knee extension phase, significantly more extension than flexion phase work was produced (95% vs 5%; p <0.001).

**Table 2:** **Concentric, eccentric and net work generated in the hip and knee joints during the propulsion and recovery phases**

	Group	P1	P2	P3	P4	Total [J]
		cc knee extensors/ ec hip flexors [J]	cc hip extensors/ ec knee flexors [J]	cc knee flexors/ ec hip extensors [J]	cc hip flexors/ ec knee extensors [J]	
Concentric work mean(SD)	P1P3	12.12(2.94)	2.03(1.17)	13.86(5.76)	1.49(1.32)	29.50‡^a^ **[** 196%]
	P1P2	20.40(9.23)	2.32(1.75)	2.95(2.11)	1.56(1.12)	27.23‡^a^ [182%]
Eccentric work mean(SD)	P1P3	-5.79(2.70)	-1.00(1.15)	-7.29(3.86)	-0.36(0.44)	-14.44 [-96%]
	P1P2	-7.96(5.42)	-0.51(0.48)	-2.65(2.78)	-1.18(1.15)	-12.30 [-82%]
Net work mean	P1P3	6.33 [42%]	1.03 [7%]	6.57 [44%]	1.13 [8%]	15.06 [100%]
	P1P2	12.44 [83%]	1.81 [12%]	0.30 [2%]	0.38 [3%]	14 93 [100%]
		Extension [J]		Flexion [J]		
Extension & Flexion phase work mean^1^	P1P3	7.36 [49%]		7.7 [51%]		15.06 [100%]
	P1P2	14.25‡^b^ [95%]		0.68 [5%]		14.93 [100%]
		Knee [J]		Hip [J]		
Net joint work mean^2^	P1P3	24.62†^c^ [163%]		-9.56 [-63%]		15.06 [100%]
	P1P2	21.66*^c^[145%]		-6.73 [-45%]		14.93 [100%]
		Active [J]		Passive [J]		
Active & Passive work mean(SD)	P1P3 & P1P2	10.37(8.52)		-4.59(5.79)		14.96(4.29)^3^

For definitions of the P1-P4 power components see Figure 1. Data is represented separately for the P1P2 and P1P3 subject groups. The percentage contributions to total net work are shown in the square brackets.

Annotations: ^**1**^Extension phase work = net P1 + net P2; Flexion phase work = net P3 + net P4; ^**2**^Net knee work = concentric knee extensor work (P1) + eccentric knee flexor work (P2) + concentric knee flexor work (P3) + eccentric knee extensor work (P4); Net hip work = eccentric hip flexor work (P1) + concentric hip extensor work (P2) + eccentric hip extensor work (P3) + concentric hip flexor work (P4); ^**3**^Total net work = Active work - Passive work. Significant differences due to ^a^concentric vs. net work; ^b^extension vs. flexion phase work; and ^c^knee vs. total work comparisons. * p < 0.01, † p < 0.005 and ‡ p < 0.001.

For the P1P3 group (Figure 4C), a large positive net power component (P1) was generated during early and middle propulsion phase. Only a small P2 occurred at ~100°, because of the weak extensor/flexor oscillations of the hip joint moment during the late propulsion phase. However a second large positive net power component (P3) occurred in the early-middle recovery phase due to the power generated by the knee flexors in excess of the power absorbed by the hip extensors. Thus the power components P1, P2, P3 and P4 contributed with 42%, 7%, 43 and 8% to total net power production, respectively, during the revolution cycle (Table 2). Significantly more (p < 0.001) concentric contraction work was produced (196%) than total net work obtained (100%). Overall positive work was produced in the knee joint, by the concentric contraction contribution of the knee extensors and flexors in P1 and P3, respectively, which significantly exceeded (163%; p < 0.005) the total net work obtained (100%), while the excess was absorbed in the hip (overall negative hip work). Since the most prominent power components P1 and P3 were localized in the propulsion (knee extension) and recovery phases (knee flexion), respectively, the extension and flexion phase work were balanced (49% and 51%), with no significant difference between the contributions (p = 0.79).

## Discussion

The main finding of the study was that the FES cycling power patterns generated by 16 subjects with SCI adhered to one of two characteristic patterns. In the most common pattern power was mainly generated from the knee extensors in the propulsion phase (12 subjects, Figure 3C). However, a quarter of the subjects generated power mainly by knee extensors in the propulsion phase and knee flexors in the recovery phase (4 subjects, Figure 4C). In both patterns the net power generation was accompanied by the occurrence of considerable amounts of eccentric power.

### Joint moments

**Figure 5:**
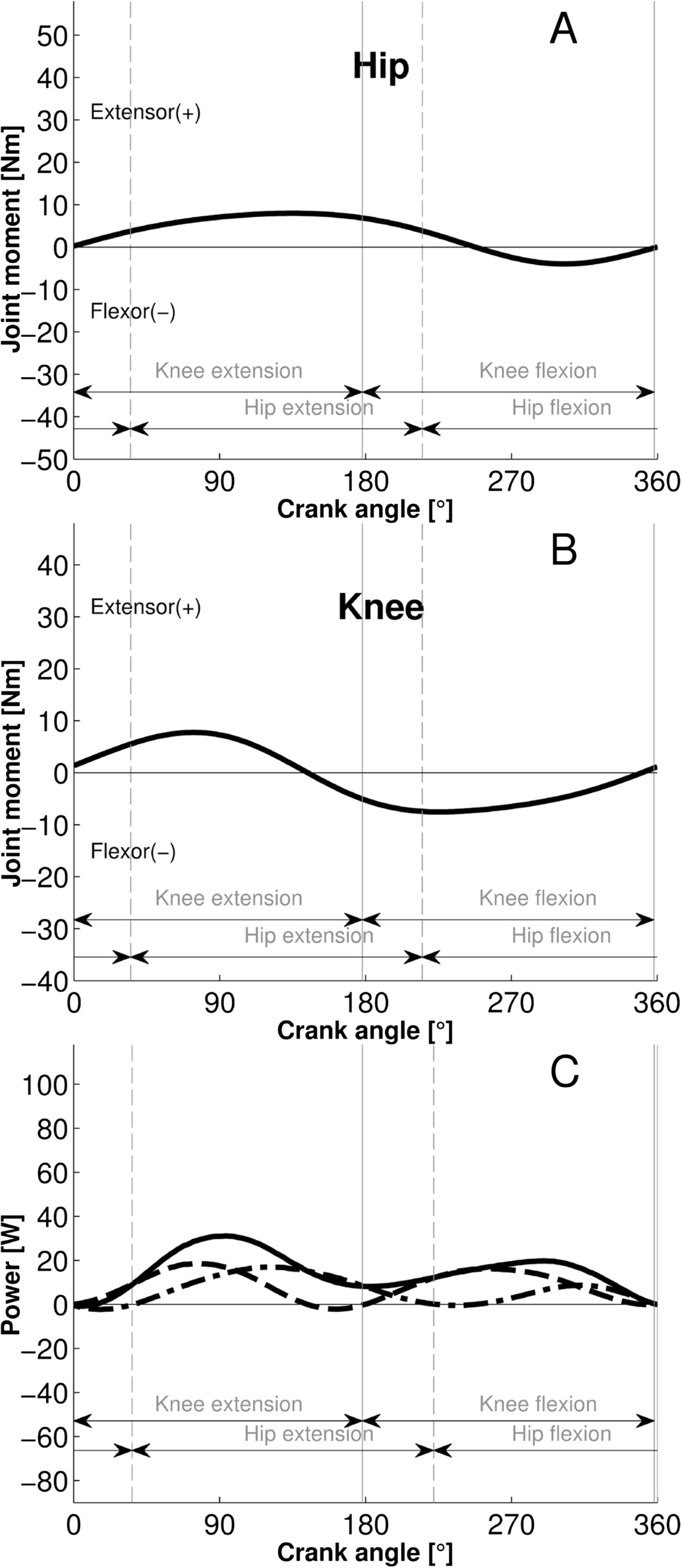
**Joint muscle moments and power in the right-leg of able-bodied subjects (n = 26), cycling volitionally at 60 rpm and a power of 15 W per leg (modified** **[**[21]**]** **).** The patterns represent averages. Panel **A** and **B**: The net hip and knee joint moment patterns. Panel **C**: The knee (dashed), hip (dashed-dotted) and net (continuous line) power patterns are displayed.

For both subject patterns, the biphasic moments of the knee, that is extensor moment during the propulsive phase and flexion moment during the recovery phase, were similar to AB subjects performing volitional recumbent cycling under similar conditions (Figure 5B), but the peak magnitudes were generally higher, except for the P1P2 recovery (Figure 3B, Figure 4B and Figure 5B).However, the biphasic hip joint moment patterns strongly contrasted with AB subjects (Figure 3A, Figure 4A and Figure 5A). While AB-subjects showed extensor moment during propulsion phase, both SCI pattern groups produced pronounced flexor but only reduced extensor moment. During recovery, the P1P2 group showed reduced extensor and flexor patterns, comparable to AB subjects (extensor around the BDC). However, the P1P3 group showed a greater extensor moment during recovery than AB subjects, which was delayed and occurred in the middle-late recovery period.

### Power distribution

The distribution of total power (95% extension phase; 5% flexion phase) for the P1P2 group (Table 2) closely confirms earlier results obtained in SCI subjects who performed mobile FES cycling [15]. This power distribution strongly contrasts to the largely balanced distribution between extension and flexion phase in AB subjects (Figure 5C). While the power distribution in AB subjects is fairly similar to the P1P3 group that producing 49% and 51% of extension and flexion phase power, respectively, the fractional contributions of the power components to extension and flexion phase power are different.

In subjects with SCI, the extension phase power was dominated by the P1 component, as the contribution of the P2 component was small or absent (Figure 3C, Figure 4C), while in AB subjects the extension phase power was distributed largely equal between P1 and P2 (Figure 5C). Whether the P2 component is present depends directly on the hamstrings strength, because this is the only muscle that can produce concentric hip extensor power in the hip- extension phase (Figure 2C). Thus, the reduced size of P2 in persons with SCI implies that the contribution of the hamstrings was reduced in the late hip-extension phase compared to AB subjects. The high variability of the hamstrings stimulation firing range compared to quadriceps [38] or a delayed increase of the contraction force due to spasticity might be responsible for low hamstring moment production in this phase.

During the recovery phase, little work was generated by the P1P2 group (small P3) compared to AB subjects due to poor knee flexor moment production from the hamstrings. However strong hamstring contractions were possible for the P1P3 group. In particular, the two subjects showing moderate and high hamstring spastics belonged to this group. The tendency of the hamstrings to develop spasticity [28], including the manifestation of inadequately high moments with FES might explain in part the occurrence of the sizeable P3 component in the P1P3 group. AB subjects produce a pronounced P4 component using hip flexors (iliopsoas) during the middle-late recovery phase of volitional recumbent cycling (Figure 5C). Since m. iliopsoas was not stimulated, only a small P4 power component due to end recovery phase stimulation of the quadriceps was found in the P1P2 and P1P3 groups.

In summary, the net cycling work was generated in subjects with SCI according to two alternative patterns depending on the hamstrings strength. If the hamstrings were weak compared to the quadriceps, the concentric knee extension work of the quadriceps generated most of net work (83%), while if the hamstrings were strong (or spastic) enough, most of net work was produced equally by the concentric knee extensor (42%) and flexor moments (44%) evoked by the quadriceps and the hamstrings, respectively. The primary concentric (positive) power generating source was the knee joint (during P1 and P3) and the hip mainly absorbed eccentric (negative) power (during P1 and P3). The findings contrast with the balanced power distribution previously found in AB subjects performing volitional cycling using the same setup, workload and cadence (P1 28%; P2 32%, P3 27% and P4 13%; [21]). The reduced P2 or P4 contributions are compensated by stronger muscle contractions in subjects with SCI.

### Co-contraction and eccentric power

In the subject shown in Figure 2C the P1 and P2 components bear considerable amounts of eccentric hip flexor and eccentric knee flexor power, produced by the quadriceps and the (sufficiently strong) hamstrings muscles, respectively. In this case a small degree of co-contraction exists, and thus eccentric power cannot be efficiently cancelled by summation of the P1 and P2 components (Additional file 1: Appendix 3). However if large co-contraction crank angle ranges of quadriceps and hamstrings exist, corresponding to overlapping stimulation firing ranges, then the summation of the P1 and P2 components results in diminishing or reducing the eccentric power . This happens by cross-cancelling: e.g. the concentric knee extensor power of P1 cancels the eccentric knee flexor power from P2 (Additional file 1: Appendix 4). However, even if sufficiently large overlapping muscle stimulation firing ranges are given, but there is a pronounced imbalance of the component magnitudes (P1 large, P2 small), the eccentric hip flexor power belonging to P1 cannot be cancelled.

The overlap (co-contraction) of the quadriceps and hamstrings stimulation firing ranges in the middle and late propulsion phase were 31 (22)° and 95 (15)° for P1P2 (Figure 3) and P1P3 (Figure 4), respectively. These correspond fairly well with previously reported crank angle ranges of quadriceps and hamstrings EMG activity overlap in AB subjects during volitional recumbent cycling [39]. Such co-contraction of the hamstrings and quadriceps muscles is known as Lombard's Paradox [40].The presented results show that power components P1 and P3 demonstrated considerable negative eccentric power in subjects with SCI (Figure 3C and Figure 4C). This strongly contrasts to the situation previously described in AB-subjects, who absorbed only small amounts of eccentric power (Figure 5C). The occurrence of substantial amounts of eccentric work assigned to P1 work can be explained by the small degree of co-contraction of the quadriceps with the hamstrings muscle that occurs because of the insufficient hamstring contraction during propulsion (small P2). Similarly, because no co-contraction of hamstrings and iliopsoas muscle exists in subjects with SCI, the eccentric power assigned to P3 could not be cancelled by P4.

An important issue is how the production of negative joint-power influences the energetic cost of movement. Kinetic methods based on inverse dynamics analysis use the work performed by hypothetical joint moment actuators as a measure of the energetic cost of movement [32, 41]. This approach assesses the muscular mechanical energy expenditure (MMEE) as the sum of positive and negative work done by the joint moments (Additional file 1: Appendix 1d). Thus for the P1P3 and the P1P2 group presenting considerable amounts of negative power (Table 2), the fractional transmission of MMEE to pedal work is 15.06 J/ (29.50 J + 14.44 J) = 0.34 and 14.93 J/(27.23 J + 12.30 J) = 0.38, respectively. This suggest that 2/3 of the MMEE is lost in subjects with SCI because of inefficient co-contraction, while for AB subjects who present only a small or negligible amount of negative power (Figure 5), the fractional transmission is via much more efficient co-contractions ~1. However, using MMEE is compromised by the presence of bi-articulate muscles and elastic energy, particularly in that it probably underestimates the biomechanical efficiency of muscle contractions during SCI FES cycling (Additional file 1: Appendix 1d).

Moreover it has been proposed [42] that co-contraction of two-joint antagonist muscles could minimize the stress cost produced in the muscles . This means for example that during cycling co-contraction of hamstrings and quadriceps to produce the P2 peak can distribute the shared workload more efficiently [39] than the quadriceps or hamstrings contracting can alone. Thus the muscular stress cost in adequately co-contracting muscles for able-bodied volitionally cycling subjects is assumed to be lower (small/absent eccentric work) than for SCI FES cycling with inefficient co-contraction (large eccentric work).

### Stimulation

The present study focused on the investigation of moment and power patterns evoked in fresh muscles by FES, avoiding decreasing magnitudes and possible change of the pattern shapes caused by fatigue [17, 43]. To prevent fatigue, an active cycling time of 60 s was selected (closely similar to [38, 39, 44], which is shorter than normal exercise session. Although fatigue is not an issue of present work, stimulation parameters for normal exercise sessions were used, that means the stimulation frequency of 30 Hz [16] and the pulse duration of 500 μs [45] were selected to minimize fatigue and possibly to maximize evoked moment, respectively. Choosing higher frequencies (>50 Hz) would have led possibly to pattern changes due to fatigue even during 60 s exercise.

The electrode placements adopted in the present study for quadriceps (rectus femoris and the three vasti) and hamstrings muscle groups (biceps femoris short and long head, semimembranosus, semitendinosus) stimulation correspond to widely used practice [28]. However the allocation of joint moments in a certain angle range to the action of a muscle group being stimulated over this range, was done under the reserve that other muscles could possibly also be co-stimulated and thus contributed to the generated joint moments. For example, it is possible that the adductor magnus might also have been co-stimulated with the hamstrings group and thus might have contributed to the hip extensor moment [46]. The present study discerns between effects of ‘quadriceps’ and ‘hamstrings’ stimulation, but further experimental and modeling work is needed to reveal which individual muscles are actually stimulated with ‘hamstrings stimulation’, and whether these muscles produce relevant joint moments.

In the present study, the gluteal muscles were not stimulated because in preliminary measurements the glutei group produced very small or not measurable moments in most subjects. The stimulation of the glutei muscle group would have supported the action of the hamstrings producing hip extensor moment during co-contraction (P2 component). While glutei moment could be evoked in some subjects, the moment generated was lower than produced by hamstrings. The variability and lack of gluteal response may be due to a high threshold of activation and the variable proximity of motor nerves to skin surface [38].

The stimulation firing angles used in this study were obtained by measuring individual static pedal force measurements [29] at all crank angle positions, and selecting the largest range of stimulation angles that produced positive crank torque for each muscle group (Additional file 1: Appendix 2). The stimulation firing ranges showed a remarkable similarity (with a maximum difference of 25°) to the crank angle ranges that produced maximum cycling power under acute conditions in simulation [47] and experimental (~5 minutes cycling) studies [38]. Thus, the requirement that high co-contraction should be achieved to optimize load sharing between the hamstrings and quadriceps appears compatible with the maximization of acute power. However wide stimulation angles during prolonged stimulation might be sub-optimal due to the higher fatigue associated with FES [48]. Future work should investigate whether fatigue changes the moment patterns and co-contraction mechanisms.

Potentially the preferential recruitment of the superficial instead of the deeper parts of the hamstrings muscles by FES in subjects with SCI could have contributed to the insufficient hamstring contraction during the second half of propulsion phase. The net hamstring work depends on the balance of the hip extensor and knee flexor moments of the hamstrings, which means it depends on the moment arms of the hamstrings group in the knee and hip joints. Thus, it is possible that by using FES which preferentially activates the more superficial muscles of the hamstrings group, comparatively higher knee/hip moment arm ratios are obtained [38], than with volitional contractions, leading to less hip extensor joint moment in subjects with SCI. Thus, the small work produced by the hamstrings could also be a consequence of the FES technology used.

## Conclusions

The majority of subjects with SCI produced the greatest proportion of FES cycling power from quadriceps knee extension. In a minority of subjects power was generated approximately equally from quadriceps knee extension and hamstring knee flexion. The co-contraction activity of antagonists is generally low in subjects with SCI because of the weakness of the hamstrings (compared to quadriceps) due to the superficial and insufficient muscle mass activation with FES and the missing iliopsoas activation. Low co-contraction however causes considerable amounts of eccentric power.

Thus clinicians are advised to use overlapping stimulation ranges and to increase hip extensor contribution by developing individual training programs for increase strength and fatigue resistance of the hamstrings and glutei muscles, rather than seeking a selective stimulation of the quadriceps to achieve high knee extensor and low hip flexor moment [49]. Additionally, future work has to investigate whether it is feasible to increase hip flexor contribution using lumbo-sacral root [10] or magnetic stimulation of the iliopsoas muscle [50].

## Electronic supplementary material

Additional file 1: Appendices. (DOC 3 MB)
