# Supplementary material for: A biomechanical cause of low power production during FES cycling of subjects with SCI
Source: J Neuroeng Rehabil. 2014 Aug 16;11:123. doi: 10.1186/1743-0003-11-123 (PMC4143553; doi:10.1186/1743-0003-11-123)
Supplement: Supplementary file 1 — Additional file 1: Appendices. (DOC 3 MB) [file 12984_2013_643_MOESM1_ESM.doc]

**Appendices**

**Appendix 1: Inverse Dynamics**

1. **Equations for inverse Dynamics**

Abbreviations used:

CG = centre of gravity;

$g=$gravitational acceleration;

$m_{fs}=$mass of the foot-shank segment;

$I_{fs}=$ inertial moment of the foot-shank segment with respect to the CG;

$r_{s1}=$distance of the foot-shank CG to the knee joint axis;

$\theta_{s1}=$angular orientation of the line defined by foot-shank CG and the knee joint axis;

$r_{s2}=$distance of the foot-shank CG to the pedal axis;

$\theta_{s2}=$angular orientation of the line defined by foot-shank CG and the pedal axis;

$m_{t}=$mass of the thigh segment;

$I_{t}=$inertial moment of the thigh segment with respect to the CG;

$r_{t1}=$distance of the thigh CG to the hip joint axis;

$r_{t2}=$distance of the thigh CG to the knee joint axis;

$\theta_{t}=$angular orientation of the thigh segment;

$a_{Xfs},a_{Yfs}=$X and Y components of the linear acceleration of the foot-shank CG with respect to the resting coordinate system;

$a_{fs}=$angular acceleration of the foot-shank segment with respect to the resting coordinate system;

$a_{Xt},a_{Yt}=$X and Y components of the linear acceleration of the thigh CG with respect to the resting coordinate system;

$a_{t}=$angular acceleration of the thigh segment with respect to the resting coordinate system;

$P_{X},P_{Y}=$X and Y components of the pedal force acting on the foot-shank;

$R_{XK},R_{YK},R_{XH},R_{YH}=$X and Y components of the reaction forces in the knee and hip joints respectively;

$M_{K},M_{H}=$knee and hip joint moments.

Based on the kinematics and external forces acting on a multi segmental rigid mechanical system, the inverse dynamics method provides the system internal joint forces and moments. In this study the one side leg with immobilized ankle was modelled as a two-dimensional, rigid, frictionless, two-body mechanical system, consisting of the thigh and the foot-shank segments with the cylindrical knee joint between them (Appendix Fig. 1). The hip joint was considered fixed. Given the segments’ kinematics ($\theta_{s1},\theta_{s2},\theta_{t},a_{Xfs},a_{Yfs},a_{fs},a_{Xt},a_{Yt},a_{t}$) and the pedal forces $(P_{X},P_{Y}),$ result the joint reaction forces $(R_{XK},R_{YK},R_{XH},R_{YH})$and the joint moments $(M_{K},M_{H})$by using free body analysis writing Newton’s equations around the CG.

For the foot-shank segment:

$$\sum Y:$$

$$P_{Y}-m_{fs}\cdot g+R_{YK}=m_{fs}\cdot a_{Yfs}$$

$$\sum X:$$

$$P_{X}+R_{XK}=m_{fs}\cdot a_{Xfs}$$

$$\sum M:$$

$$\begin{matrix} M_{K}-R_{XK}\cdot r_{s1}\cdot\sin\theta_{s1}-R_{YK}\cdot r_{s1}\cdot\cos\theta_{s1}+ \\ +P_{Y}\cdot r_{s2}\cdot\cos\theta_{s2}+P_{X}\cdot r_{s2}\cdot\sin\theta_{s2}=I_{fs}\cdot a_{fs} \end{matrix}$$

For the thigh segment:

$$\sum Y:$$

$$-R_{YK}-m_{t}\cdot g+R_{YH}=m{}_{t}\cdot a{}_{Yt}$$

$$\sum X:$$

$$-R_{XK}+R_{XH}=m{}_{t}\cdot a{}_{Xt}$$

$$\sum M:$$

$$\begin{matrix} M_{H}-M_{K}-R_{YH}\cdot r_{t1}\cdot\cos\theta_{t}+R_{XH}\cdot r_{t1}\cdot\sin\theta_{t}- \\ -R_{XK}\cdot r_{t2}\cdot\sin\theta_{t}-R_{YK}\cdot r_{t2}\cdot\cos\theta_{t}=I_{t}\cdot a_{t} \end{matrix}$$

Only the joint moments were further used in the present study.

1. **Net joint moment and power obtained by inverse dynamics**

The inverse dynamic method finds the net joint moment which is the algebraic (signed) sum of all extensor and flexor moments acting in the joint. Due to the indeterminacy problem , the inverse mechanics method cannot distinguish between the individual extensor/flexor moment contributions from muscles and ligaments which span the same joint and together produce the resulting net moment. For example a resulting 1 Nm net extensor moment may be the difference between a 2 Nm and a 1 Nm moment or between a 12 Nm and an 11 Nm moment; each of these would presumably have a different metabolic cost.

However, the inverse mechanics method provides moments for each joint separately and so it can formally account for power contributions of different joints to the total power. Thus it is possible that 15 W total power is produced in a leg by generating +20 W concentric (positive) power in the knee and absorbing -5 W eccentric (negative) power in the hip. Or the 15 W total power could be produced by generating +8 and +7 W in the knee and the hip joint respectively. Again, different metabolic cost would be expected in each case.

1. **Power balance equation and muscle elastic energy**Using the net joints moments obtained by inverse dynamics, the instantaneous power equation for a leg is :

$\frac{dE_{l}}{dt}=P_{H}+P_{K}+F_{p}\cdot v_{p}$.

The mechanical energy of the leg $E_{_{l}}$ accounts for gravitational potential and kinetic and energy of the modelled segments, but it does not account for the elastic energy of muscles, tendons or other structures. Instead net joint moments ($M_{H} and M_{K}$) and net joint power $(P_{H}=M_{H}\cdot\omega_{H}; P{}_{K}=M_{K}\cdot\omega_{K} )$ contain not only the joint moment and power contributions of muscle contractile structures, but also of those originating from elastic structures. It is known, for example, that negative work done on the elastic tendons can be regained as positive work (stretch -contraction cycle) contributions. Elastic contributions to net joint moment may be quite considerable at low workload (e.g. 30W).
Assuming that muscle elasticity is developed from the muscle tension produced by stimulation, neither the inverse dynamic method, nor the subtraction of passive moments used in this study can separate the moment contributions into muscle contractile and muscle elastic moments. A comparison of directly computed pedal work with the sum of joint powers obtained by inverse dynamics cannot help, because the joint moments were obtained by inverse dynamics calculations from the pedal forces. To discern power from contractile and elastic elements of individual muscles would require reliable, detailed muscle models which is almost impossible to construct because of uncertainties with respect to the intrinsic properties of the individual muscles .

Thus the instantaneous pedal power$F_{p}\cdot v_{p}$is generated by net joint power (including elasticity) and the decrease of the mechanical energy of the leg:

$P_{H}+P_{K}-\frac{dE_{l}}{dt}=F_{p}\cdot v_{p}$.
Integrating over the movement cycle and considering that the mechanical energy (potential gravitational and kinetic) for the leg system over one crank revolution cycle is$\Delta E_{l}=0$, we can obtain the pedal work:

$W_{H}+W_{K}=\int_{cycle} F_{p}\cdot v_{p}\cdot dt$.

Thus pedal work can be found by adding the work at the hip (positive or negative) and the work at the knee (positive or negative) over the cycle. This is true regardless of whether muscles have elasticity or not. If they do, the joint moments $M_{H} and M_{K}$ and the joint work $W_{H}=\int_{cycle} M_{H}\cdot\omega_{H}\cdot dt$ and $W_{K}=\int_{cycle} M_{K}\cdot\omega_{K}\cdot dt$ contain elastic components too. If it is reasonable to assume, that the elastic energy has no change over the cycle, then joint work $W_{H} and W_{K}$ over the cycle represent only contractile muscular work, and pedal work will be given by their sum.

1. **Estimation of the energetic cost of cycling**

Based on inverse dynamics calculations it is reasonable to assess the energetic cost of cycling movement by using the muscular mechanical energy exchange (MMEE) defined as the absolute sum of positive and negative work done at the joints by the net moments ()
$MMEE=\int_{cycle} \left|P_{H}\right|\cdot dt+\int_{cycle} \left|P_{K}\right|\cdot dt$
This definition contains important assumptions which are not unconditionally fulfilled. Because the MMEE is calculated from the absolute value of the joint power, the sources are considered not to be intercompensated and recuperative . However, biarticular muscles can convert the negative energy absorbed at one joint to positive energy generated at the other joint, so intercompensation takes place. Additionally recuperation of elastic energy occurs when muscles are shortening immediately after lengthening. Thus it is assumed, that MMEE overestimates the energetic costs.

A**ppendix 2. Stimulation angle ranges**

The mean FES firing angles used in current study were obtained from static crank torque measurements [5]. App. Tab. 1 compares the current angles to those used in a previous study conducted by Sinclair to maximize acute power.

|  | | Current study^*^ Static torque maximisation | Sinclair study  Power maximisation |
| --- | --- | --- | --- |
| Quadriceps | Start | 350 ± 6 | 326 |
|  | Stop | 153 ± 16 | 143 |
| Hamstrings | Start | 93 ± 35 | 71 |
|  | Stop | 265 ± 23 | 245 |

**Appendix Table 1.** Firing angles used during the current and a previous study. *The current study angles were advanced by 50° (60 rpm x 0.84°/rpm) to compensate for muscle force rise times.

**Appendix 3: Joint moment and power patterns from a small overlap in stimulation.**
The joint moment and power patterns from a study subject that showed a small overlap of quadriceps and hamstrings stimulation ranges are represented in Fig. 2. To demonstrate the combined effect of quadriceps and hamstrings stimulation, for this subject the joint moment and power patterns were also obtained from the separate stimulation of the quadriceps and hamstrings (Appendix Fig. 2 & 3). Note that due to the effect of fatigue, a perfect linear superposition of moments and power could not be obtained with separate stimulation, thus only qualitative conclusions can be drawn from the data. Fig. 2C shows that if only a small overlap of hamstring and quadriceps stimulation occurs then the effect of moment summation is small. Thus, the power component P1 with combined stimulation (Fig. 2C) is mainly produced by the quadriceps contraction (Appendix Fig. 2C) and the combined P2 and P3 are produced by the hamstrings contraction (Appendix Fig. 3C).

**Appendix 4: Joint moment and power patterns from a large overlap in stimulation.**
The joint moment and power patterns from a study subject that showed a large overlap of quadriceps and hamstrings stimulation ranges are represented in Appendix Fig. 4. To demonstrate the combined effect of quadriceps and hamstrings stimulation, for this subject the joint moment and power patterns were also obtained from the separate stimulation of the quadriceps and hamstrings (Appendix Fig. 5 & 6). Note that due to the effects of fatigue, a perfect linear superposition of moments and power could not be obtained with separate stimulation, thus only qualitative conclusions can be drawn from the data.

When stimulated separately, the quadriceps and hamstrings moments produced across the hip joint overlap in the crank angle range 60-90°. The magnitude of the hamstrings moment is comparable to that of the quadriceps moment in this angular range (circle arrows in Appendix Fig. 5A & 6A). Thus, the combined action of the muscles reduces the flexor moment produced across the hip joint in this crank angle range (circle arrow Appendix Fig. 4A). Correspondingly, the concentric extensor power produced by the hamstrings in the hip (circle arrow in Appendix Fig. 6C) partially cancels the eccentric flexor power produced by the quadriceps in the hip (circle arrow in Appendix Fig. 5C), reducing the eccentric hip flexor power in the early-middle propulsion phase (circle arrow Appendix Fig. 4C). Thus, the net work of P1 will increase as if the knee extensor power were increased. Therefore, power cancellation in the early-middle propulsion phase means that the quadriceps (rectus femoris) muscle transfers power produced in the hip by the hamstrings to the knee joint .

In the middle-late propulsion phase, the concentric knee extensor power from the quadriceps (Appendix Fig. 5C) fully cancels the eccentric knee flexor contribution from the hamstrings (Appendix Fig. 6C). Thus, the hamstrings muscle transfers power generated in the knee via the quadriceps muscle to the hip joint .

**Appendix Figures**

**
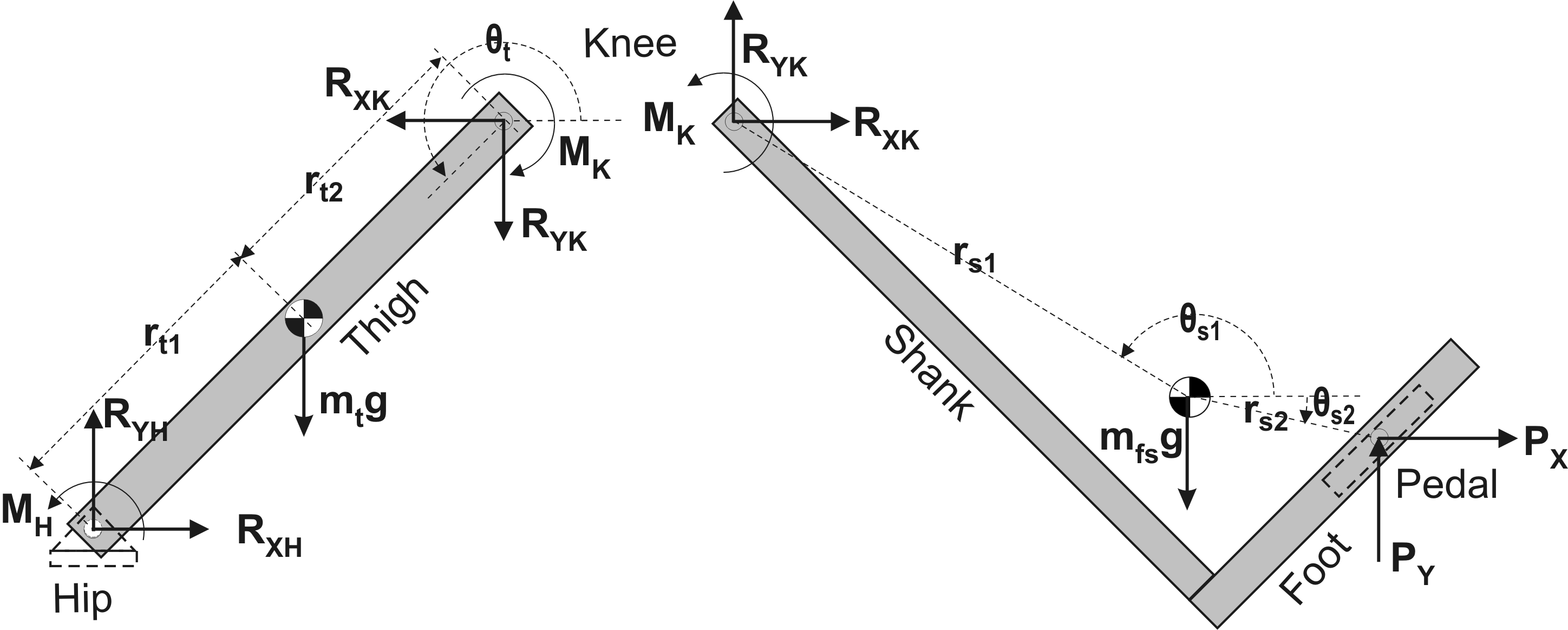
**

**Appendix Figure 1.** Two-segment one-side rigid body model of recumbent cycling with ankle immobilization. For notations see Appendix 1a.

**
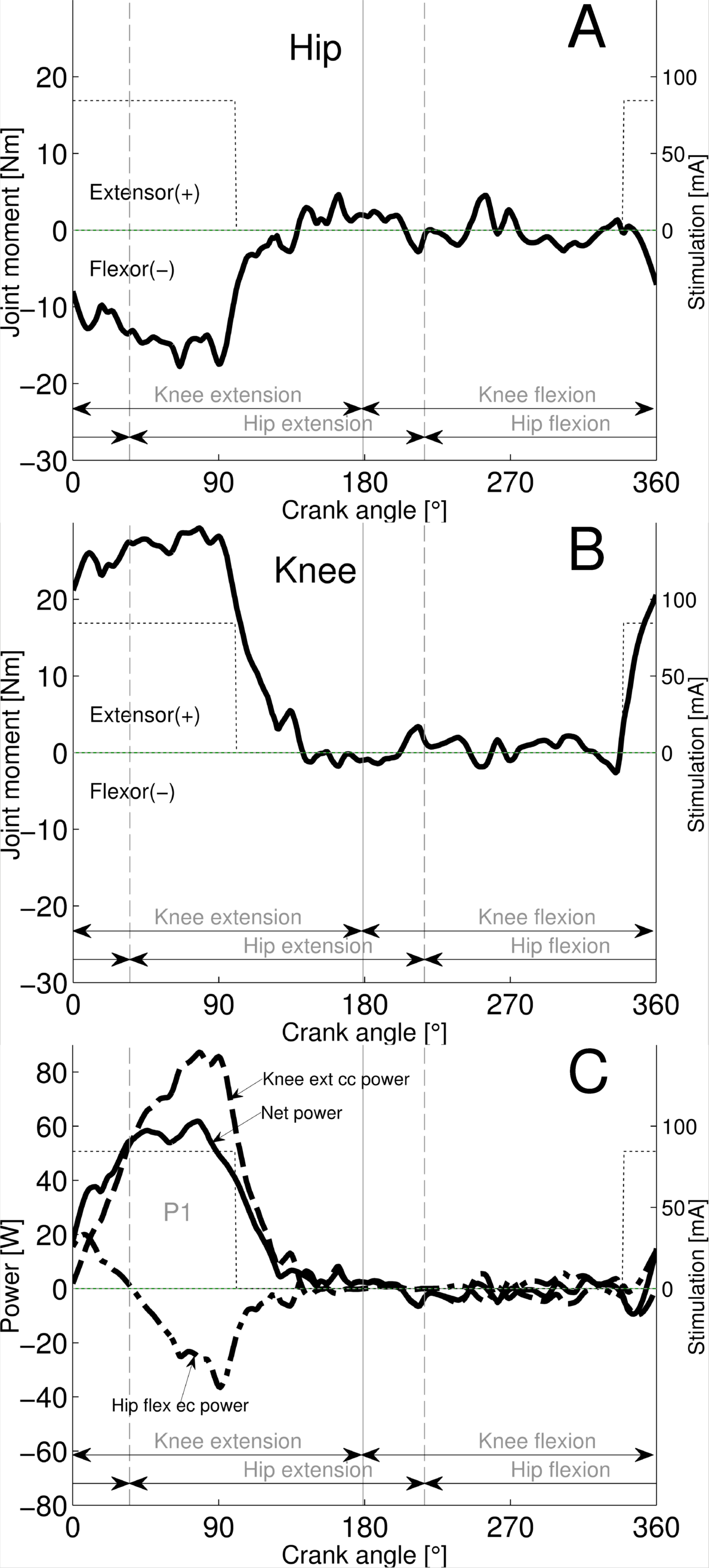
**

**Appendix Figure 2.** Joint muscle moments and power produced with quadriceps stimulation in the right leg of the representative subject shown in Fig. 2. Active pedalling was performed with motor support at a constant cadence of 60 rpm at the same stimulation level as in Fig. 2. Panel A and B: The hip and knee joint moment patterns. Panel C displays the knee (dashed), hip (dashed-dotted) and the net power (continuous) patterns. The power component P1 is marked. The intensity for quadriceps stimulation (thin black dotted) is an on-off step-function. The stimulation is phase advance corrected by 50°.


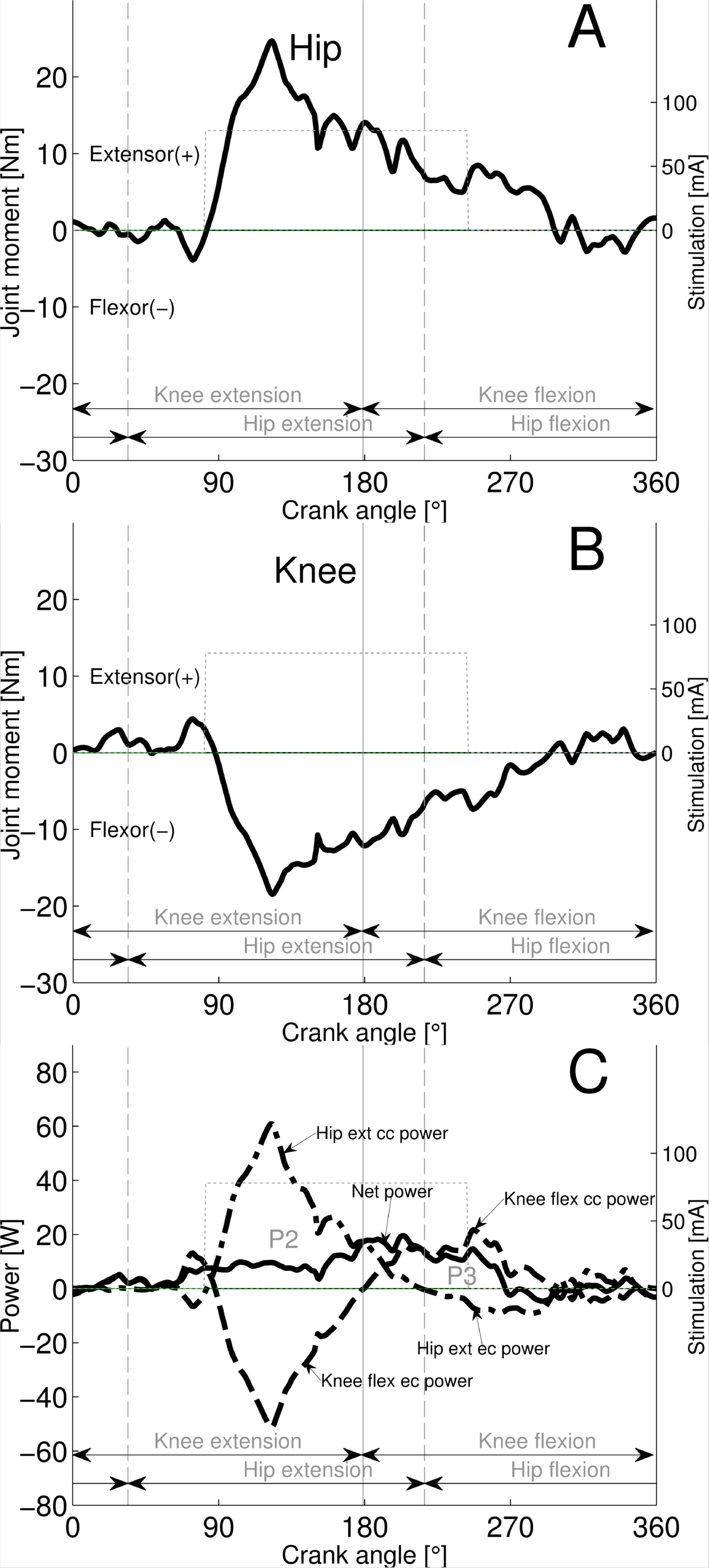
 **Appendix Figure 3**. Joint muscle moments and power produced with hamstrings stimulation in the right leg of the representative subject shown in Fig. 2. Active pedalling was performed with motor support at a constant cadence of 60 rpm at the same stimulation level as in Fig. 2. Panel A and B: The hip and knee joint moment patterns. Panel C displays the knee (dashed), hip (dashed-dotted) and the net power (continuous) patterns. The power components P2 and P3 are marked. The intensity for hamstrings stimulation (thin grey dotted) stimulation is an on-off step-function. The stimulation is phase advance corrected by 50°.


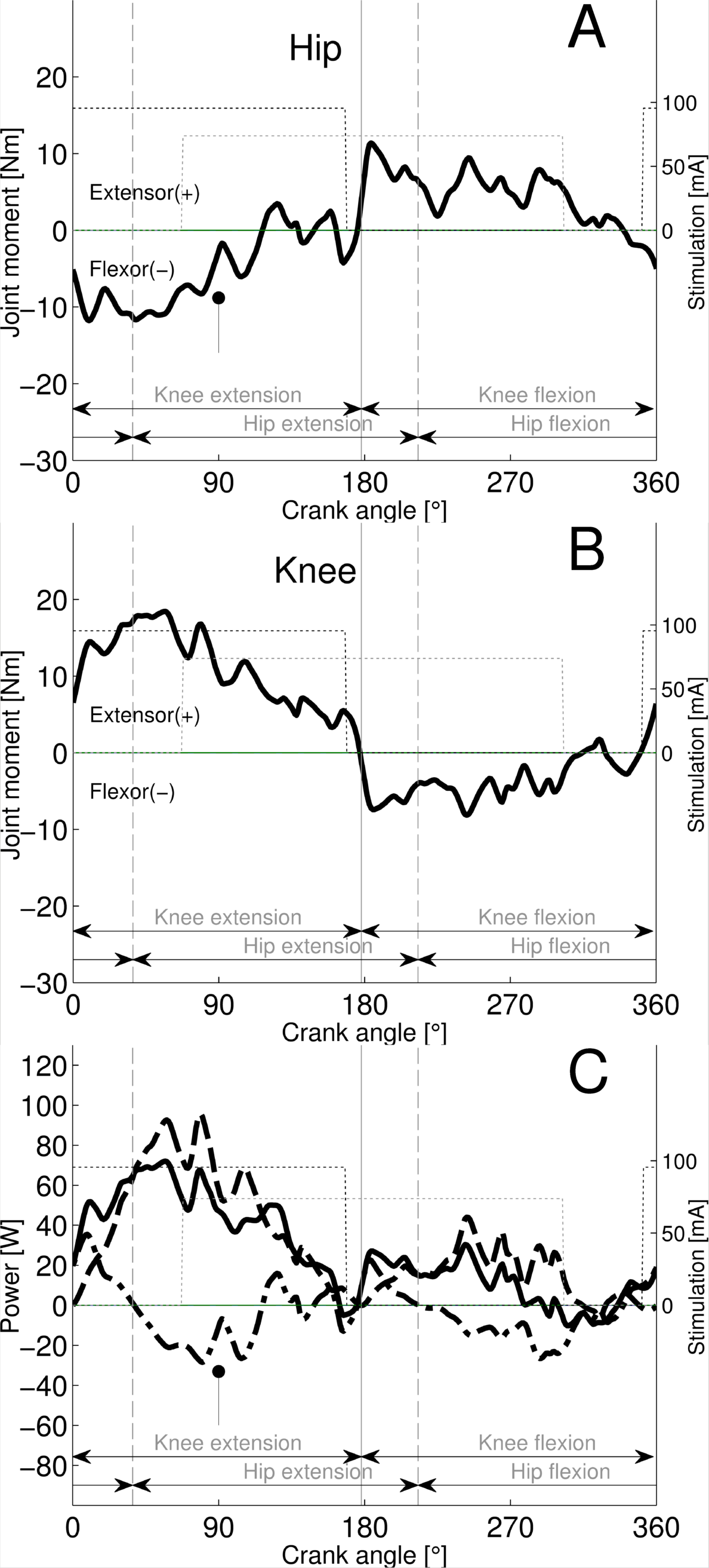
 **Appendix Figure 4**. The joint muscle moments and power measured from the right leg of a representative subject that displayed a large overlap in quadriceps and hamstrings contraction during FES pedalling. Active pedalling was performed at 60 rpm and 15 W per leg. Panel A and B: The hip and knee joint moment patterns. Panel C displays the knee (dashed), hip (dashed-dotted) and the net power (continuous) patterns. The circle arrows point to an example of antagonistic torque summation and power cancelation at the hip (compare Appendix Fig. 4, 5 & 6). The intensities for quadriceps stimulation (thin black dotted) and hamstrings (thin grey dotted) are on-off step-functions. The stimulations are phase advance corrected by 50°.


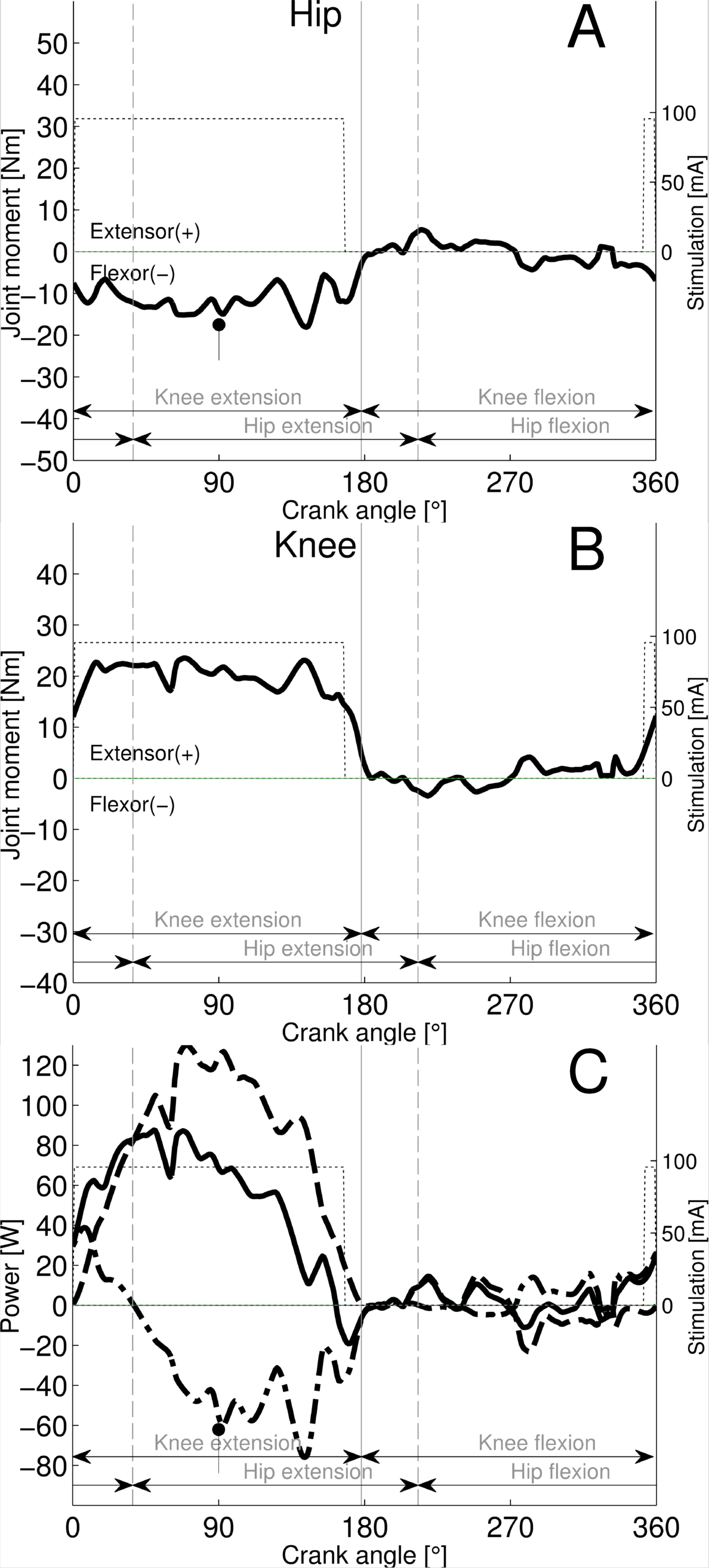


**Appendix Figure 5**.
The joint muscle moments and power produced with only quadriceps stimulation in the right leg of the subject represented in Appendix Fig. 4. Active pedalling was performed with motor assistance at 60 rpm at the same stimulation level as in Appendix Fig. 4. Panel A and B: The hip and knee joint moment patterns. Panel C displays the knee (dashed), hip (dashed-dotted) and net power (continuous) patterns. The intensity for quadriceps stimulation (thin black dotted) is an on-off step-function. The stimulation is phase advance corrected by 50°.

The circle arrows point to an example of antagonistic torque summation and power cancelation at the hip (compare Appendix Fig. 4, 5 & 6).


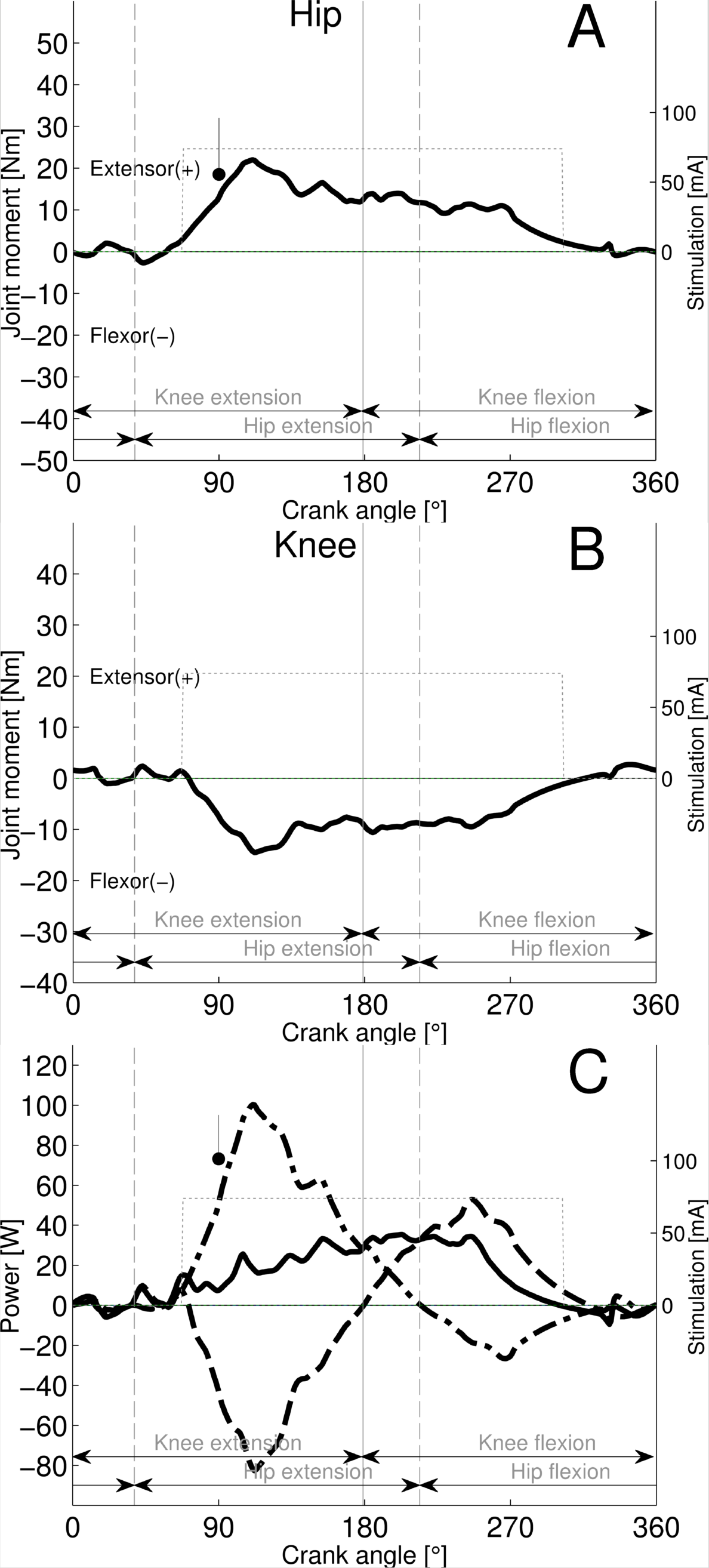


**Appendix Figure 6**
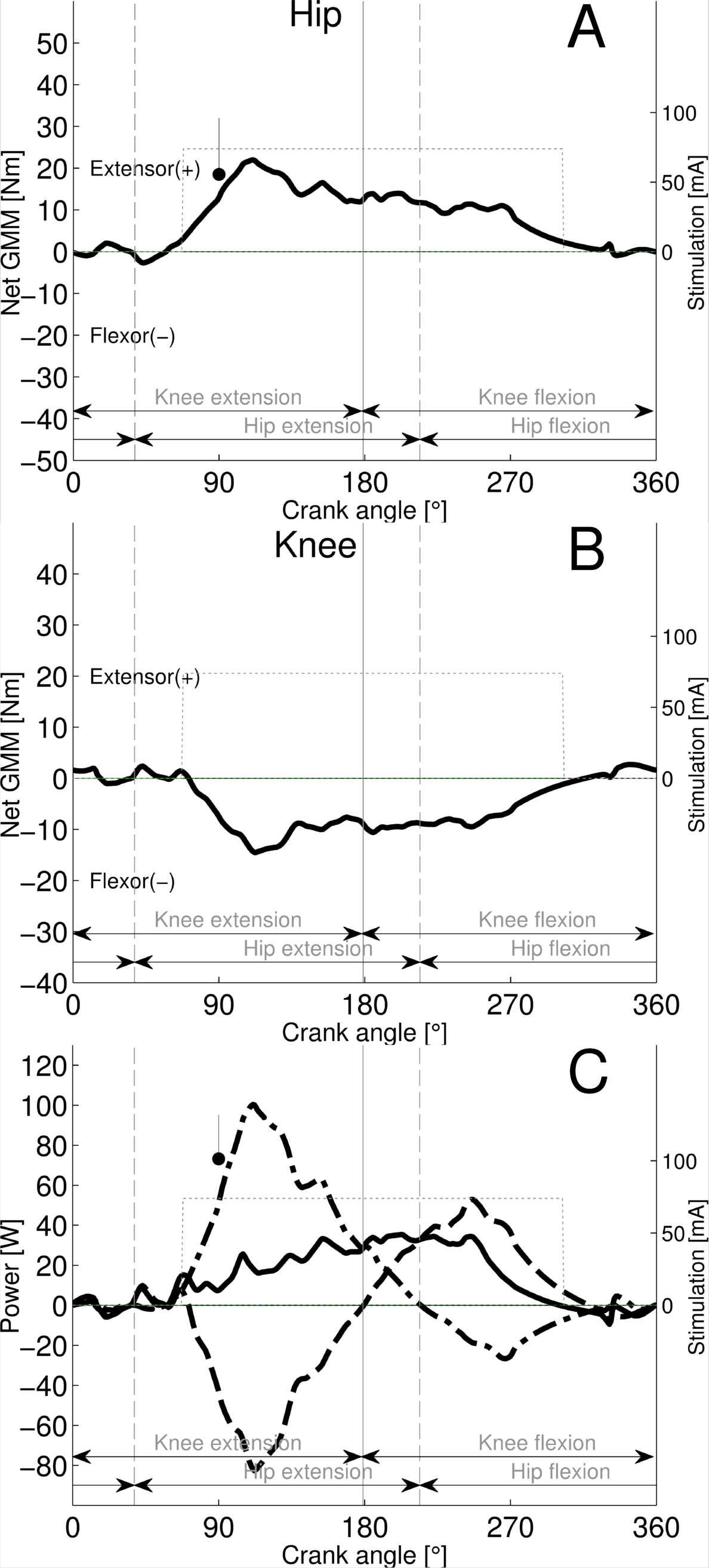
.
The joint muscle moments and power produced with only hamstrings stimulation in the right leg of the subject represented in Appendix Fig. 4. Active pedalling was performed with motor assistance at 60 rpm at the same stimulation level as in Appendix Fig. 4. Panel A and B: The hip and knee joint moment patterns. Panel C displays the knee (dashed), hip (dashed-dotted) and net power (continuous) patterns. The intensity for hamstrings stimulation (thin grey dotted) is an on-off step-function. The stimulation is phase advance corrected by 50°. The circle arrows point to an example of antagonistic torque summation and power cancelation at the hip (compare Appendix Fig. 4, 5 & 6).

**Appendix Literature**

1. Winter DA: *Biomechanics and Motor Control of Human Movement.* second edn. Waterloo: John Wiley & Sons, Inc; 1990.

2. van Ingen Schenau GJ, van Woensel WW, Boots PJ, Snackers RW, de Groot G: **Determination and interpretation of mechanical power in human movement: application to ergometer cycling.** *European journal of applied physiology and occupational physiology* 1990, **61:**11-19.

3. Kautz SA: **Biomechanics of pedalling with non-circular chainrings in cycling.** University of California Davies, Biomedical engineering; 1992.

4. Neptune RR, van den Bogert AJ: **Standard mechanical energy analyses do not correlate with muscle work in cycling.** *Journal of biomechanics* 1998, **31:**239-245.

5. Aleshinsky SY: **An energy 'sources' and 'fractions' approach to the mechanical energy expenditure problem--I. Basic concepts, description of the model, analysis of a one-link system movement.** *Journal of biomechanics* 1986, **19:**287-293.

6. Aleshinsky SY: **An energy 'sources' and 'fractions' approach to the mechanical energy expenditure problem--II. Movement of the multi-link chain model.** *Journal of biomechanics* 1986, **19:**295-300.

7. Sinclair PJ: **Forward dynamic modelling of cycling for people with spinal cord injury.** The University of Sydney, School of Exercise and Sport Science, Faculty of Health Sciences; 2001.

8. van Ingen Schenau GJ, Cavanagh PR: **Power equations in endurance sports.** *Journal of biomechanics* 1990, **23:**865-881.
